# Alkaline Peptone Water-Based Enrichment Method for *mcr-3* From Acute Diarrheic Outpatient Gut Samples

**DOI:** 10.3389/fmed.2018.00099

**Published:** 2018-05-03

**Authors:** Qiaoling Sun, Yanyan Hu, Hongwei Zhou, Lingbin Shu, Hanyu Wang, Zixian Huang, Rong Zhang

**Affiliations:** ^1^Department of Clinical Laboratory, Second Affiliated Hospital of Zhejiang University, School of Medicine, Hangzhou, China; ^2^University of Connecticut, Storrs, CT, United States; ^3^The Affiliated High School of Hangzhou Normal University, Hangzhou, China

**Keywords:** *mcr-3*, *Aeromonas veronii*, diarrheic outpatient, alkaline peptone water, enrichment method

## Abstract

A third plasmid-mediated colistin resistance gene, *mcr-3*, is increasingly being reported in Enterobacteriaceae and *Aeromonas* spp. from animals and humans. To investigate the molecular epidemiology of *mcr* in the gut flora of Chinese outpatients, 152 stool specimens were randomly collected from outpatients in our hospital from May to June, 2017. Stool specimens enriched in alkaline peptone water or Luria-Bertani (LB) broth were screened for *mcr-1, mcr-2*, and *mcr-3* using polymerase chain reaction (PCR)-based assays. Overall, 19.1% (29/152) and 5.3% (8/152) of the stool samples enriched in alkaline peptone water were PCR-positive for *mcr-1* and *mcr-3*, respectively, while 2.7% (4/152) of samples were positive for both *mcr-1* and *mcr-3*. Strains isolated from the samples that were both *mcr-1*- and *mcr-3*-positive were subjected to antimicrobial susceptibility testing by broth microdilution. They were also screened for the presence of other resistance genes by PCR, while multilocus sequence typing and whole-genome sequencing were used to investigate the molecular epidemiology and genetic environment, respectively, of the resistance genes. *mcr-3*-positive *Aeromonas veronii* strain 126-14, containing a *mcr-3*.*8*-*mcr-3*-like2 segment, and *mcr-1*-positive *Escherichia coli* strain 126-1, belonging to sequence type 1485, were isolated from the sample from a diarrheic butcher with no history of colistin treatment. *A. veronii* 126-14 had a colistin minimum inhibitory concentration (MIC) of 2 µg/mL and was susceptible to antibiotics in common use, while *E. coli* 126-1 produced TEM-1, CTX-M-55, and CTX-M-14 β-lactamases and was resistant to colistin, ceftazidime, and cefotaxime. Overall, there was a higher detection rate of *mcr-3*-carrying strains with low colistin MICs from the samples enriched in alkaline peptone water than from samples grown in LB broth.

## Introduction

Since the identification of a third plasmid-mediated colistin resistance gene, *mcr-3*, in a porcine *Escherichia coli* isolate from China in 2017 (1), several *mcr-3* variants have been detected in clinical *E. coli* and *Salmonella* isolates from Denmark, Spain, and China (2–5). The amino acid sequence of MCR-3 is highly similar to that of phosphoethanolamine transferases from various *Aeromonas* and Enterobacteriaceae species (1). Ling et al. reported that chromosomally located *mcr-3* variants, including *mcr-3.3* and *mcr-3*-like, which were identified in *Aeromonas veronii* from chicken meat, showed 95.2 and 84.2% nucleotide sequence identity, respectively, to *mcr-3* from *E. coli* of porcine origin (6). Interestingly, the reported minimum inhibitory concentration (MIC) of colistin for the *mcr-3*-carrying *A. veronii* isolate from chicken meat was 2 µg/mL while colistin MICs for the *mcr-3*-positive Enterobacteriaceae were in the range of 4–8 µg/mL. Thus, *mcr-3*-positive *Aeromonas* spp. strains are likely to go undetected by routine clinical tests. Our previous studies have established an optimized enrichment method for the screening of *mcr-1* from human gut and environmental water sources (7, 8), in which the *mcr-1-*carrying strains demonstrated MICs for colistin of 1–32 µg/mL. As *Aeromonas* spp. generally prefer an alkaline pH, we improved the enrichment method using alkaline peptone water. In this study, we used the newly developed enrichment method to investigate the epidemiology of *mcr* in the gut flora of outpatients treated in our hospital.

## Materials and Methods

### Stool Specimens and Microbial Enrichment

A total of 152 stool specimens were randomly collected from outpatients suffering from acute diarrhea at the Intestinal Clinic of the Second Affiliated Hospital of the Zhejiang University School of Medicine from May to June 2017. Aliquots (~1 g) of each stool sample were individually inoculated into 5 mL of alkaline peptone water (Binhe, Hangzhou, China) and 5 mL of Luria-Bertani (LB) broth for enrichment overnight at 35°C. The alkaline peptone water was adjusted to a pH of 8.4–9.2 and contained 15.0 g/L of tryptone, 4.0 g/L of beef extract, and 10.0 g/L of NaCl.

### Detection of *mcr*-Positive Isolates by Enrichment Culture

Following incubation, each enrichment culture tube was inverted 10 times to resuspend the cells and a 1-mL aliquot of suspension was transferred to a fresh 1.5-mL tube. The suspension was centrifuged for 3 min at 8,000 rpm, after which the supernatant was discarded and 1 mL of 0.9% (w/v) saline was added to wash and resuspend the cell pellet. The centrifugation step was then repeated, and 70 µL of ultra-pure water was added to the pellet, which was then boiled for 5 min. Following centrifugation, a 3-µL aliquot of the supernatant was used as template for polymerase chain reaction (PCR) amplification of *mcr-1, mcr-2*, and *mcr-3* as described previously (1, 9, 10).

Following initial PCR-based screening, four of the alkaline peptone water enrichment cultures tested positive for both *mcr-1* and *mcr-3* and were therefore selected for colony isolation. A 10-µL aliquot of suspension from the enrichment cultures was inoculated onto *Salmonella*–*Shigella* agar plates and incubated at 37°C overnight. Resultant colonies were selected for further purification and confirmation of the presence of the *mcr* genes using the PCR-based method described above. Final identification of the *mcr-*positive colonies was performed by matrix-assisted laser desorption/ionization time-of-flight mass spectrometry (Bruker Daltonik GmbH, Bremen, Germany) analysis. As this method cannot distinguish between *E. coli* and *Shigella* spp., Kligler Iron Agar and Motility-indole-Urea medium were added to help identify *E. coli* strains. An *mcr-3-*positive *A. veronii* isolate and a *mcr-1-*positive *E. coli* isolate were identified from one of the four *mcr-1*- and *mcr-3*-positive alkaline peptone water enrichment cultures, and were tested for antimicrobial susceptibility and screened for the presence of other common β-lactamase-encoding genes using further PCR.

### Antimicrobial Susceptibility Testing

The MICs of eight antibiotics against *mcr-*positive isolates were determined using a broth microdilution procedure. The susceptibilities of each of the isolates to meropenem, ceftazidime, cefotaxime, cefoperazone–sulbactam, amikacin, and ciprofloxacin were determined according to the Clinical and Laboratory Standards Institute guidelines (11). The breakpoints for colistin and tigecycline against *E. coli* were obtained from the European Committee on Antimicrobial Susceptibility Testing breakpoint tables (12). *E. coli* ATCC25922 was used as a quality control strain for broth microdilution assays.

### Detection of Other Common β-Lactamase-Encoding Genes

Additional β-lactamase-encoding genes, including *bla*_TEM_, *bla*_SHV_, *bla*_CTX-M-1-group_, and *bla*_CTX-M-9-group_ were detected by PCR using previously described primers and conditions (13).

### Multilocus Sequence Typing (MLST)

Molecular typing of the *mcr-1-*positive *E. coli* isolate was performed by MLST using conditions and primers described on the MLST website (http://mlst.warwick.ac.uk/mlst/dbs/Ecoli). The sequences of the seven housekeeping genes were compared with those in the *E. coli* MLST database.

### Whole-Genome Sequencing

The selected *mcr-3-*positive *A. veronii* isolate was submitted for 300-bp paired-end whole-genome sequencing using the Illumina Hiseq 2500 system (Annoroad, Beijing, China). The raw Illumina reads were assembled into a draft genome sequence using CLC Genomics Workbench 9.0 (CLC Bio, Aarhus, Denmark). Antibiotic resistance genes were analyzed using SRST2 (14), with reference sequences for the antibiotic resistance genes obtained from the ARG-ANNOT database (15).

## Results

### Detection of *mcr*-Positive Isolates Following Enrichment Culture

Following enrichment in alkaline peptone water, 19.1% (29/152) and 5.3% (8/152) of the stool samples were PCR-positive for *mcr-1* and *mcr-3*, respectively, while 18.4% (28/152) of the samples enriched in LB broth were positive for *mcr-1*. None of the LB enrichment samples tested positive for *mcr-3* (Table 1), and none of the samples from either enrichment method were positive for *mcr-2*.

**Table 1:** Initial polymerase chain reaction screening results for the presence of *mcr* genes in enrichment stool cultures.

Enrichment culture type	Sample size	*mcr-1*(*n*, %)	*mcr-2*(*n*, %)	*mcr-3*(*n*, %)	*mcr-1* + *mcr-3*(*n*, %)
Alkaline peptone water	152	29, 19.1	0, 0	8, 5.3	4, 2.7
Luria-Bertani broth	152	28, 18.4	0, 0	0, 0	0, 0

An *mcr-3-*positive *A. veronii* isolate (strain 126-14) and a *mcr-1-*positive *E. coli* isolate (strain 126-1) were simultaneously isolated from the same alkaline peptone water-enriched stool sample. The sample was collected from a 42-year-old male pork butcher with no medication history of colistin. He was admitted to the gastroenterology clinic for 2 days suffering from acute abdominal pain and diarrhea following ingestion of watermelon. He developed a fever (38.4°C), and stool analysis showed the yellow loose stool did not contain any leukocytes or erythrocytes. Levofloxacin and viable *Lactobacillus acidophilus* tablets were administrated, and the patient attained complete remission.

### Antimicrobial Susceptibility Testing

Identified as sequence type 1485 by MLST, *mcr-1-*positive *E. coli* isolate 126-1 showed resistance to colistin, ceftazidime, and cefotaxime, and additional PCR analyses confirmed the co-existence of *bla*_TEM-1_, *bla*_CTX-M-55_, and *bla*_CTX-M-14_ in this strain. *mcr-3*-positive *A. veronii* isolate 126-14 was susceptible to all tested antibiotics, and had MICs for colistin and tigecycline of 2 and 1 µg/mL, respectively (Table 2).

**Table 2:** MICs and resistance gene profiles of *mcr-3*-positive *Aeromonas veronii* 126-14 and *mcr-1*-positive *Escherichia coli* 126-1.

Isolate	MICs of (μg/mL)								Resistance gene(s)
	CL	MEM	CAZ	CTX	SCF	AMK	CIP	TIG	
*A. veronii* 126-14	2	≤0.25	≤0.5	≤0.25	≤1/0.5	≤8	≤0.25	1	*mcr-3*
*E. coli* 126-1	8	≤0.25	>32	>32	8/4	≤8	0.5	1	*mcr-1, bla*_TEM-1_, *bla*_CTX-M-55_, *bla*_CTX-M-14_

*MIC, minimum inhibitory concentration; CL, colistin; MEM, meropenem; CAZ, ceftazidime; CTX, cefotaxime; SCF, cefoperazone-sulbactam; AMK, amikacin; CIP, ciprofloxacin; TIG, tigecycline*.

### Whole-Genome Sequencing

**Figure 1:**
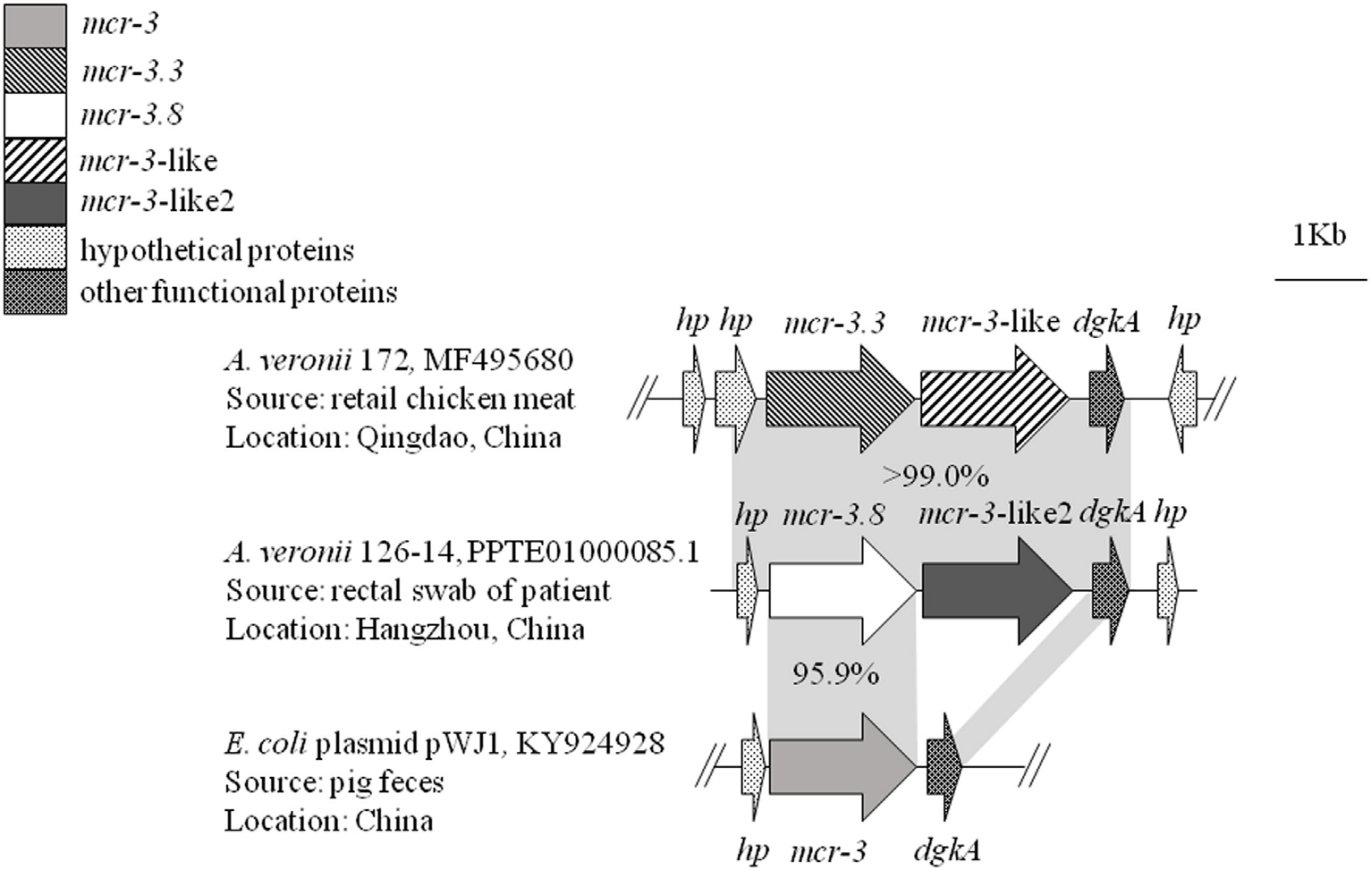
The genetic environment of the *mcr-3.8-mcr-3-*like2 segment in the *Aeromonas veronii* isolate identified in this study. Arrows represent the directions of the genes. Gray shading indicates two areas with significant similarity.

**Figure 2:**
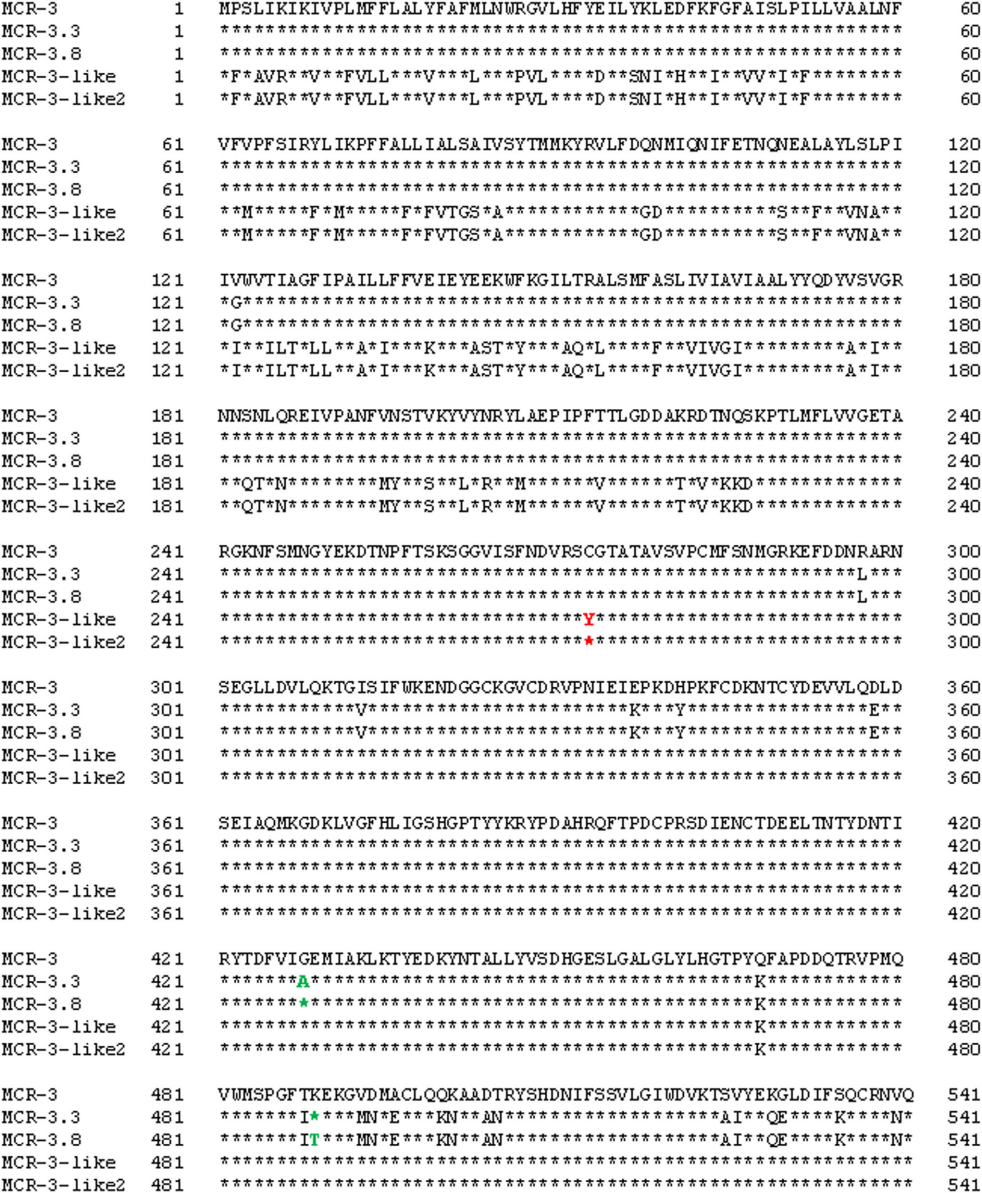
Alignment of the MCR-3, MCR-3.3, MCR-3-like, MCR-3.8, and MCR-3-like2 sequences from *Escherichia coli* (GenBank accession no. KY924928) and *Aeromonas* isolates (GenBank accession no. MF495680 and PPTE01000085.1).

Whole-genome sequencing of *mcr-3* positive *A. veronii* isolate 126-14 produced 146 contigs. Two adjacent *mcr-3* variants, the novel upstream variant termed *mcr-3.8* and the downstream variant termed *mcr-3*-like2, were located on 5,338-bp contig 85 and were separated by only 66 bp. The sequences of these two variants have been deposited in GenBank under accession no. PPTE01000085.1. Both variants showed >99.0% nucleotide and amino acid sequence identity to the *mcr-3.3* and *mcr-3-like* genes in *A. veronii* isolated from chicken meat, and 95.9 and 87.2% nucleotide sequence identity and 95.8 and 84.8% amino acid sequence identity, respectively, to the original *mcr-3* gene (Figures 1 and 2). Similar to the *mcr-3.3-mcr-3-*like segment in a previously identified *A. veronii* isolate (GenBank accession no. MF495680), the *mcr-3.8-mcr-3-*like2 segment in *A. veronii* 126-14 was surrounded by both hypothetical genes and diacylglycerol kinase alpha-encoding gene *dgkA* but lacked transfer elements (Figure 1).

## Discussion

To the best of our knowledge, this study is the first report of the co-occurrence of a *mcr-3*-positive *A. veronii* isolate and a *mcr-1*-positive *E. coli* isolate from the gut of a diarrheic outpatient. Gram-negative *Aeromonas* spp. cause various infections in both humans and animals, with *Aeromonas*-associated diarrhea and gastroenteritis the most frequent manifestations of infection in humans. *Aeromonas* infections affect all age groups, including both healthy and immunocompromised individuals (16). The transferable colistin resistance gene *mcr-1* has been reported in Enterobacteriaceae isolated both from food-producing animals and humans, with carriage rates of 5.1 and 6.2%, respectively (17, 18). These carriage rates indicate that the emergence and spread of *mcr-1* probably occurred first in isolates of animal origin, which then spread to humans. In this study, the *mcr-3*-positive *A. veronii* strain isolated from the fecal sample might have been the cause of the acute diarrhea. The patient was employed as a pork butcher and had no history of colistin use, which indicated that the *mcr-3* and the *mcr-1* genes likely originated from isolates of food–animal origin. This suggests that foodstuffs of farm animal origin may act as a critical transmission vehicle in the dissemination of mobile colistin resistance genes from animal-associated with human-associated bacteria.

In this study, 19.1% (29/152) and 5.3% (8/152) of the stool samples enriched in alkaline peptone water were PCR-positive for *mcr-1* and *mcr-3*, respectively, while only 18.4% (28/152) of the LB broth-enriched samples tested positive for *mcr-1*. This suggests that the alkaline peptone water enrichment step before direct sample testing resulted in higher sensitivity of the *mcr* screening from human stool samples compared with LB broth cultivation. In addition, the detection rate of *mcr-1* in this study (approximately 19%) was much higher than that reported previously (about 6%) (18), probably due to the enrichment step and direct enriched sample testing by PCR. Furthermore, *mcr-3*-positive *A. veronii* isolate 126-14 had a colistin MIC of 2 µg/mL, which was in agreement with the previously reported colistin MIC for a *mcr-3*-carrying *A. veronii* isolate from chicken meat (6). Therefore, these results suggest that unsupplemented *Salmonella*–*Shigella* agar rather than medium supplemented with colistin is better for selection of *mcr-3*-carrying *Aeromonas* spp. strains. Overall, only one *mcr-3*-positive *A. veronii* isolate was recovered from the four PCR-positive alkaline peptone water-enriched stool samples. Although *Aeromonas* spp. are widely distributed in soil (19), foodstuffs (20), and aquatic environments (21), they usually constitute a small percentage of the human gut flora (22). As such, the number of *Aeromonas* spp. present in an enrichment culture may still be too low to detect *via* direct culture plating methods.

In conclusion, the alkaline peptone water enrichment method was optimal for detection of *mcr-3*-carrying strains with low colistin MICs from the human gut microbiota. The method is simple to perform and can be used in any laboratory that is equipped to perform PCR assays and can obtain alkaline peptone water at the proper pH. In addition, as the human intestine may serve as a reservoir for antibiotic resistance genes, including *mcr-3*, and play an important role in horizontal gene transfer, the rapid horizontal spread of mobile colistin resistance genes between and within Enterobacteriaceae and *Aeromonas* spp. in the human gut should be closely monitored.

## Ethics Statement

Written informed consent was obtained from the patient for the publication of this research. The Ethics Committee of the Second Affiliated Hospital of Zhejiang University, School of Medicine, already approved this work.

## Author Contributions

RZ and HZ designed and supervised the study. HW participated in stool sample collecting. QS, LS, HW, and ZH conducted the microbial enrichment, PCR, antimicrobial susceptibility testing, and strain characterization. YH analyzed the whole-genome sequencing. RZ, QS, YH, HZ, and LS contributed to the data interpretation and manuscript writing. All the authors have approved the final version and have agreed to be accountable for all aspects of the work.

## Conflict of Interest Statement

The authors declare that the research was conducted in the absence of any commercial or financial relationships that could be construed as a potential conflict of interest.

## References

[1] Yin WJ Li H Shen YB Liu ZH Wang SL Shen ZQ Novel plasmid-mediated colistin resistance gene mcr-3 in *Escherichia coli*. mBio (2017) 8(3):e543–517. 10.1128/mBio.00543-17 28655818 PMC5487729

[2] Roer L Hansen F Stegger M Sönksen UW Hasman H Hammerum AM. Novel mcr-3 variant, encoding mobile colistin resistance, in an ST131 *Escherichia coli* isolate from bloodstream infection, Denmark, 2014. Euro Surveill (2017) 22(31):30584. 10.2807/1560-7917.ES.2017.22.31.30584 28797324 PMC5553058

[3] Hernández M Iglesias MR Rodríguez-Lázaro D Gallardo A Quijada NM Miguela-Villoldo P Co-occurrence of colistin-resistance genes mcr-1 and mcr-3 among multidrug-resistant *Escherichia coli* isolated from cattle, Spain, September 2015. Euro Surveill (2017) 22(31):30586. 10.2807/1560-7917.ES.2017.22.31.30586 28797328 PMC5553059

[4] Litrup E Kiil K Hammerum AM Roer L Nielsen EM Torpdahl M Plasmid-borne colistin resistance gene mcr-3 in salmonella isolates from human infections, Denmark, 2009–17. Euro Surveill (2017) 22(31):30587 10.2807/1560-7917.ES.2017.22.31.30587 28797325 PMC5553060

[5] Liu L Feng Y Zhang XX McNally A Zong ZY A new variant of mcr-3 in an extensively drug-resistant *Escherichia coli* clinical isolate carrying mcr-1 and blaNDM-5. Antimicrob Agents Chemother (2017) 61(12):1757–1717. 10.1128/AAC.01757-17 PMC5700326 28971871

[6] Ling ZR Yin WJ Li H Zhang QD Wang XM Wang Z Chromosome-mediated mcr-3 variants in *Aeromonas veronii* from chicken meat. Antimicrob Agents Chemother (2017) 61:1272–1217. 10.1128/AAC.01272-17 28848017 PMC5655048

[7] Hu YY Wang YL Sun QL Huang ZX Wang HY Zhang R Colistin resistance gene mcr-1 in gut flora of children. Int J Antimicrob Agents (2017) 50:593–7. 10.1016/j.ijantimicag.2017.06.011 28668691

[8] Zhou HW Zhang T Ma JH Fang Y Wang HY Huang ZX Occurrence of plasmid- and chromosome-carried mcr-1 in waterborne Enterobacteriaceae in China. Antimicrob Agents Chemother (2017) 61:e00017–17. 10.1128/AAC.00017-17 28559252 PMC5527621

[9] Liu YY Wang Y Walsh TR Yi LX Zhang R Spencer J Emergence of plasmid-mediated colistin resistance mechanism MCR-1 in animals and human beings in China: a microbiological and molecular biological study. Lancet Infect Dis (2016) 16(2):161–8. 10.1016/S1473-3099(15)00424-7 26603172

[10] Xavier BB Lammens C Ruhal R Kumar-Singh S Butaye P Goossens H Identification of a novel plasmid-mediated colistin-resistance gene, mcr-2, in *Escherichia coli*, Belgium, June 2016. Euro Surveill (2016) 21(27). 10.2807/1560-7917.ES.2016.21.27.30280 27416987

[11] Clinical and Laboratory Standards Institute (CLSI). Performance Standards for Antimicrobial Susceptibility Testing. M100. 27th ed Wayne, PA: (2017). Available online at: https://clsi.org/standards/products/microbiology/documents/m100/ (Accessed: July, 2017).

[12] European Committee on Antimicrobial Susceptibility Testing (EUCAST). Breakpoint Tables for Interpretation of MICs and Zone Diameters. Version 7.1. Växjö (2017). Available online at: http://www.eucast.org/clinical_breakpoints/ (Accessed: July, 2017).

[13] Yu YS Ji SJ Chen YG Zhou WL Wei ZQ Li LJ Resistance of strains producing extended-spectrum beta-lactamases and genotype distribution in China. J Infect (2007) 54(1):53–7. 10.1016/j.jinf.2006.01.014 16533535

[14] Inouye M Dashnow H Raven LA Schultz MB Pope BJ Tomita T SRST2: rapid genomic surveillance for public health and hospital microbiology labs. Genome Med (2014) 6(11):90. 10.1186/s13073-014-0090-6 25422674 PMC4237778

[15] Gupta SK Padmanabhan BR Diene SM Lopez-Rojas R Kempf M Landraud L ARG-ANNOT, a new bioinformatic tool to discover antibiotic resistance genes in bacterial genomes. Antimicrob Agents Chemother (2014) 58(1):212–20. 10.1128/AAC.01310-13 24145532 PMC3910750

[16] Silva LCAD Leal-Balbino TC Melo BST Mendes-Marques CL Rezende AM Almeida AMP Genetic diversity and virulence potential of clinical and environmental *Aeromonas* spp. isolates from a diarrhea outbreak. BMC Microbiol (2017) 17(1):179. 10.1186/s12866-017-1089-0 28821241 PMC5563053

[17] Yang YQ Li YX Song T Yang YX Jiang W Zhang AY Colistin resistance gene mcr-1 and its variant in *Escherichia coli* isolates from chickens in China. Antimicrob Agents Chemother (2017) 61(5):e1204–16. 10.1128/AAC.01204-16 28242671 PMC5404584

[18] Zhong LL Phan HTT Shen C Doris-Vihta K Sheppard AE Huang X High rates of human fecal carriage of mcr-1-positive multi-drug resistant Enterobacteriaceae isolates emerge in China in association with successful plasmid families. Clin Infect Dis (2018) 66(5):676–85. 10.1093/cid/cix885 29040419 PMC5848316

[19] Goswami G Deka P Das P Bora SS Samanta R Boro RC Diversity and functional properties of acid-tolerant bacteria isolated from tea plantation soil of Assam. 3 Biotech (2017) 7(3):229. 10.1007/s13205-017-0864-9 28681289 PMC5498365

[20] Gowda TK Reddy VR Devleesschauwer B Zade NN Chaudhari SP Khan WA Isolation and seroprevalence of *Aeromonas* spp. Among common food animals slaughtered in Nagpur, Central India. Foodborne Pathog Dis (2015) 12(7):626–30. 10.1089/fpd.2014.1922 25946095

[21] Chenia HY Duma S. Characterization of virulence, cell surface characteristics and biofilm-forming ability of *Aeromonas* spp. isolates from fish and sea water. J Fish Dis (2017) 40(3):339–50. 10.1111/jfd.12516 27425219

[22] Igbinosa IH Igumbor EU Aghdasi F Tom M Okoh AI. Emerging *Aeromonas* species infections and their significance in public health. ScientificWorldJournal (2012) 2012:625023. 10.1100/2012/625023 22701365 PMC3373137

